# Investigating cognitive reserve, symptom resolution and brain connectivity in mild traumatic brain injury

**DOI:** 10.1186/s12883-023-03509-8

**Published:** 2023-12-20

**Authors:** Natascha Ekdahl, Marika C. Möller, Catharina Nygren Deboussard, Britt-Marie Stålnacke, Marianne Lannsjö, Love Engström Nordin

**Affiliations:** 1 Centre for Research and Development, Uppsala University/ County Council of Gävleborg, Gävle, Sweden; 2 Department of Clinical Sciences, Karolinska Institutet, Stockholm, Sweden; 3 Department of Rehabilitation Medicine, Danderyd University Hospital, Stockholm, Sweden; 4 Department of Community Medicine and Rehabilitation, Rehabilitation Medicine, Umeå University, Umeå, Sweden; 5 Department of Neuroscience, Rehabilitation Medicine, Uppsala University, Uppsala, Sweden; 6 Department of Neurobiology, Care Sciences and Society (NVS), Division of Clinical Geriatrics, Karolinska Institutet, Stockholm, Sweden; 7 Department of Diagnostic Medical Physics, Karolinska Institutet, Stockholm, Sweden

**Keywords:** Fatigue, Fatigability, Cognition, Imaging, Neuropsychology

## Abstract

**Background:**

A proportion of patients with mild traumatic brain injury (mTBI) suffer long-term consequences, and the reasons behind this are still poorly understood. One factor that may affect outcomes is cognitive reserve, which is the brain's ability to maintain cognitive function despite injury. It is often assessed through educational level or premorbid IQ tests. This study aimed to explore whether there were differences in post-concussion symptoms and symptom resolution between patients with mTBI and minor orthopedic injuries one week and three months after injury. Additional aims were to explore the relationship between cognitive reserve and outcome, as well as functional connectivity according to resting state functional magnetic resonance imaging (rs-fMRI).

**Method:**

Fifteen patients with mTBI and 15 controls with minor orthopedic injuries were recruited from the emergency department. Assessments, including Rivermead Post-Concussion Questionnaire (RPQ), neuropsychological testing, and rs-fMRI scans, were conducted on average 7 days (SD = 2) and 122 days (SD = 51) after injury.

**Results:**

At the first time point, significantly higher rates of post-concussion symptoms (U = 40.0, *p* = 0.003), state fatigue (U = 56.5, *p* = 0.014), and fatigability (U = 58.5, *p* = 0.025) were observed among the mTBI group than among the controls. However, after three months, only the difference in post-concussion symptoms remained significant (U = 27.0, *p* = 0.003). Improvement in post-concussion symptoms was found to be significantly correlated with cognitive reserve, but only in the mTBI group (Spearman’s rho = -0.579, *p* = .038). Differences in the trajectory of recovery were also observed for fatigability between the two groups (U = 36.5, *p* = 0.015). Moreover, functional connectivity differences in the frontoparietal network were observed between the groups, and for mTBI patients, functional connectivity differences in an executive control network were observed over time.

**Conclusion:**

The findings of this pilot study suggest that mTBI, compared to minor orthopedic trauma, is associated to both functional connectivity changes in the brain and concussion-related symptoms. While there is improvement in these symptoms over time, a small subgroup with lower cognitive reserve appears to experience more persistent and possibly worsening symptoms over time. This, however, needs to be validated in larger studies.

**Trial registration:**

NCT05593172. Retrospectively registered.

## Introduction

Although the majority of individuals who sustain a mild traumatic brain injury (mTBI) recover within a few weeks, as many as one third continue to experience persistent symptoms, such as fatigue, headaches, and depression [1, 2]. Identifying individuals at risk of long-term consequences after mTBI early on is crucial for organizing suitable follow-up and treatment. However, this is challenging due to, among others, the diversity and nonspecific nature of the symptoms [3, 4]. In emergency settings, computerized tomography (CT) scans are a commonly used method for assessing the grade of brain injury. Unfortunately, this method has been proven inadequate in detecting the subtle alterations that occur in the brain after mTBI and has a low prognostic value for risk of developing post-concussion syndrome [5].

Due to the limitations of conventional neuroimaging techniques in detecting subtle alterations that could have a significant impact on prognosis and long-term outcomes following mTBI, functional magnetic resonance imaging (fMRI) has emerged as a more sensitive biomarker for evaluating post-mTBI outcomes [5–7]. Resting state functional magnetic resonance imaging (rs-fMRI) is a noninvasive technique that can measure functional connectivity by examining the blood oxygen level-dependent (BOLD) signal of different brain regions while the individual is at rest. Using rs-fMRI, abnormal connectivity, including both hypo- and hyperconnectivity has been identified in several resting state networks after mTBI [8]. A review summarizing the main findings from both rs-fMRI and fMRI studies revealed that abnormal increases in thalamocortical activity, alterations in the default mode network (DMN) and changed activity in the prefrontal cortex are common findings after mTBI [5]. In the DMN, the precuneus area seems to be particularly susceptible to the effects of brain injury [5]. Current research has mainly focused on identifying abnormalities in the subacute phase of rs-fMRI after mTBI, but there is a lack of studies including fMRI data, that have followed subjects over time [9, 10].

Furthermore, it has been proposed that when studying the connection between fMRI biomarkers and mTBI outcomes, additional variables like demographics and cognitive measures should be considered alongside imaging biomarkers [3]. A variable linked to demographic status and cognition that has been shown to be important for outcome in brain injuries or pathology is cognitive reserve [11]. Cognitive reserve is the ability of the brain to maintain cognitive function in the face of brain injury or disease and is thought to be influenced by factors such as education, occupation, and leisure activities [12]. Studies have shown that individuals with higher levels of cognitive reserve may be better able to compensate for brain injury and maintain cognitive function in conditions ranging from Alzheimer’s disease, MS, and mTBI [13–15].

Using healthy controls for comparison of functional connectivity changes in mTBI may not be ideal as they often are self-selected and have not experienced the psychological effects associated with a trauma. Orthopedic controls, individuals who have sustained non-brain injuries but are otherwise similar to mTBI patients in terms of age, sex, and comorbidities, have been proposed as a more appropriate comparison group for mTBI studies [16].

Statistically significant differences in trait fatigue and fatigability between patients with mTBI and an orthopedic control group 1 week after injury have been presented in a previous article based on data from baseline measures stemming from this patient group [17]. The aim of the current study was thus to investigate whether there were differences in post-concussion symptoms and symptom resolution between patients with mTBI and minor orthopedic injuries one week and three months after injury. Additional aims were to explore the relationship between cognitive reserve and outcome, as well as functional connectivity according to resting state functional magnetic resonance imaging (rs-fMRI).

## Materials and methods

The study was conducted between January 2015 and April 2016 and is a prospective controlled observational fMRI study. Patients were recruited from Danderyd University Hospital’s emergency department, and all evaluations, including MRI scans, were performed on-site at the hospital. The study design has previously been described in detail in Clinical Trials Protocol NCT05593172.

### Participants

From the emergency department at Danderyd University Hospital 15 patients and 15 controls were recruited. For the patient group, consecutive patients with mTBI significant enough to warrant a CT scan was included. The control group (OC) consisted of orthopedic patients with minor traumatic injuries to the hand, arm, foot or leg with no need for surgical intervention. The patients in both groups were between 18 and 40 years of age and were included in the same manner and during the same time frame.

A total of 99 individuals declined participation, 17 with mTBI and 82 with orthopedic injuries, primarily citing time constraints and inconvenience as reasons for non-participating. Of the non-participants, 88% with mTBI and 64% with orthopedic injuries were men. There was no significant difference in age between participators and non-participators. At follow-up two individuals from both groups were lost.

The WHO Collaborating Center of Neurotrauma Task Force criteria were used to define mTBI, which specifies a transient neurological deficit, a Glasgow Coma Scale (GCS, [18]) score between 13–15 but no more than 30 min loss of consciousness and 24 h post-traumatic amnesia [19].

Exclusion criteria were as follows:

- Uncertain duration of loss of consciousness
- Contraindications to magnetic resonance imaging (MRI),
- Previously acquired brain injury,
- A progressive neurological disorder or another injury/illness with short expected survival
- Dependence on help in daily living before the current injury
- Severe visual impairment
- Non-Swedish speaking

### Procedure

Eligible study participants were contacted 1–3 days after injury. All participants underwent assessment at two time points: the subacute phase (T1, mean 7 days after injury, range 2–12 days), and follow-up (T2, mean 122 days after injury, range 81–324 days). Injury-related data, including GCS score upon arrival at the emergency department and brain CT scan results, was extracted from medical records. During the first assessment demographic data and data regarding prior medical and psychiatric history was obtained, using a structured interview. rs-fMRI were performed at both time points. To reduce drop-out rates, the timeframe for the second assessment was extended.

### MRI data acquisition protocol

Brain imaging was performed with a 3.0T Philips Ingenia MRI (Philips, Best, The Netherlands) equipped with an 8-channel head coil. Three structural imaging sequences were included: 1) T1-weighted anatomical images using a 3-dimensional gradient echo sequence with a field of view, FOV, 250 × 250 mm, matrix 227 × 227, slice thickness 1.2 mm, slice gap 0.6 mm, number of slices 301, echo time, TE, 3.58ms, repetition time, TR 7.69ms). 2) T2-FLAIR (fluid-attenuated inversion recovery); FOV 230 × 230 mm, matrix 234 × 234, slice thickness 4 mm, slice gap 0.4 mm, number of slices 37, TE = 125 ms; TR = 11000 ms, inversion time, TI, 2800 ms for suppression of water signal for better lesion detection. 3) T2-weighted FFE (fast field echo) images; FOV 230 × 183 mm, matrix 256 × 205, slice thickness 4 mm, slice gap 1 mm, number of slices 30, TE 16 ms, TR 500 ms. The BOLD resting-state functional MRI protocol consisted of a gradient echo-planar sequence with FOV 230 × 230 mm, matrix 96 × 96, slice thickness 4 mm, TE = 35 ms, TR = 3000 ms, flip angle = 90°, and voxel size of 2.4 × 2.4x4 mm. The acquisition time was 8 min and the total number of volumes acquired were 160. Patients were instructed to keep their eyes closed, to think about nothing in particular and not to fall asleep.

### Instruments

#### Self-assessment

- *The Rivermead Post-Concussion Symptoms Questionnaire* (RPQ, [20]) was employed to assess self-reported symptoms. The RPQ has 16 items that assess various symptoms such as memory problems, headache, and irritation, with each item score ranging from 0 to 4. A score of 1 signifies that the symptom had previously been present, but is no longer an issue, while scores between 2 and 4 indicate present symptom severity ranging from mild to severe. In the current article the total sum was calculated, but not including when the participants scored 1, since symptoms were measured at two time points. As fatigue is a common symptom after brain injury the item that measures state fatigue (RPQ-F), was also analyzed separately.
- *The Fatigue Severity Scale* (FSS, [21]) was used to measure self-rated trait fatigability. The FSS comprises 9 questions and operates on a 7 point Likert scale A higher score indicates more fatigue. Widely used in chronic illness contexts, it is recognized for its strong psychometric properties [22].
- *The Hospital Anxiety and Depression Scale* (HADS, [23, 24]) was used to assess depression and anxiety. The scale includes separate scales for each condition. Scores range from 0 to 21, where of 8–10 indicate potential cases of depression/anxiety, and scores from 11 and above suggest more definite cases of depression/anxiety.

#### Neuropsychological assessment

- *The Swedish Lexical Decision Test* was employed to evaluate the premorbid intelligence level. The test requires participants to judge if a word is real or a non-existing word and is based on the association between word knowledge and cognitive functioning, which tend to remain stable even after a traumatic brain injury [25]. Studies have shown that The Swedish Lexical Decision Test accounts for 48% of the variance in full-scale intelligence quote measured by the WAIS [25].
- *The Digit Symbol Substitution Test/Coding* (DSST),from the Wechsler Adult Intelligence Scales (WAIS) [26] was used to assess fatigability. The fatigability measure (DSST-f) was calculated by deducting the score from the initial 60 s of the test from the score from the last 60 s. The performance time is 120 s in total. A negative score suggests increased fatigability [27]. Previous research has confirmed DSST-f as a sensitive measure for fatigability in patients with mTBI [28]. In the present study parallel versions of the test were used at the two time points to minimize practice effects.

### Analysis

Descriptive statistics used to depict demographics, injury characteristics, results on neuropsychological tests and psychological screening instruments were computed using the Jamovi statistical software [29]. A significance level of 0.05 was used for all statistical tests. Differences in demographic characteristics between groups were analyzed with Student’s t-test for parametric data and the Mann–Whitney test for nonparametric data. The difference over time between groups was calculated by subtracting T1 from T2 for each patient (d-values) and then comparing the groups using Mann–Whitney test. Improvement over time was examined using the Wilcoxon rank sum test. The Holm-Bonferroni test was employed on behavioral data where multiple comparisons were conducted. Corrections were conducted separately for the self-assessed questionnaires and the objective 6measures. For measures of effect size, r was used, where 0.1 is considered a small effect size, 0.3 medium, and 0.5 large. To analyze the relationship between recovery over time and cognitive reserve, correlation analysis with Spearman’s rho was used.

The resting-state fMRI data sets were preprocessed using FSL (http://www.fmrib.ox.ac.uk/fsl). The initial 5 timeframes in each data set were excluded to ensure temporal stability. Motion correction was performed using McFLIRT, an intra-modal motion correction tool applying rigid body transformations to correct fMRI data for motion [30]. The time curves for translational and rotational motion were assessed for all participants and data sets with motion that exceeds 1 voxel size (2,4mm) were removed from the sample. Non-brain tissue was removed using the Brain Extraction Tool (BET) [31]. To minimize noise, spatial smoothing with a 5 mm full width at half maximum (FWHM) was applied. Spatial normalization to the standard MNI brain template was performed using a 12-parameter affine transformation and mutual-information cost function. During the affine transformation the imaging data was re-sampled to isotropic resolution using a Gaussian kernel with 4 mm FWHM. Independent component analysis (ICA) is a model free and multivariate method of performing group analysis of resting state fMRI data. The ICA analysis in the present study was performed using the FSL tool MELODIC version 3.15 [32] and the multisession temporal concatenation approach. ICA was performed on all participants to obtain as good statistics as possible for the independent components. The set of spatial maps from the group-average analysis was used to generate subject-specific versions of the spatial maps, and associated time series, using dual regression [33]. First, for each subject, the group-average set of spatial maps is regressed into the subject's 4D space–time dataset. This results in a set of subject-specific time series, one per group-level spatial map. Next, those time series are regressed into the same 4D dataset, resulting in a set of subject-specific spatial maps, one per group-level spatial map. We then tested for group differences and time effects using FSL's *randomise* permutation-testing tool [34]. To correct for multiple comparisons in the group analysis 10000 permutations were performed using randomize. Contrast matrices were created in FSL´s general linear model tool to perform two-way analysis of group and time.

## Results

### Demographic and clinical characteristics

There were no significant group differences in age (mTBI M = 25.1 SD = 6.5, OC M = 27.5 SD = 7.4) or gender (7 women in the mTBI group compared to 11 in the OC group). No significant difference between the mTBI group and the OC group was found regarding length of education, estimated premorbid IQ or results on HADS screening for anxiety and depression, see Table 1. The most common cause of injury for the mTBI group was falling accidents and for the OC group it was sports accidents. For further details on the cause of injury see the previously published article [17]. In the mTBI group one patient had a GCS of 14, and all the others had a score of 15. Two patients had small pathological findings on CT of the brain. Neither required surgery nor did their results differ from the other patients in the mTBI group regarding self-assessment measures or neuropsychological tests. No statistically significant difference was found between patients with mTBI and the OC group regarding time between the injury and assessments (baseline and follow-up). Due to within group variation in time to follow-up Spearman’s rho was calculated for the time between injury and assessment compared to the rate of improvement for the RPQ, RPQ-F and DSST-f. There was no significant correlation between time to follow-up and improvement on any of the measures either for the groups separately or taken together. In the mTBI group 8 patients reported a history of previous concussion (i.e. trauma to the head but does not necessarily qualify as mTBI) with no reported sequelae compared to 3 patients in the OC group. Two patients in the mTBI group and one in the OC group reported psychiatric comorbidity (anxiety and depression). Two patients in both groups reported medical comorbidities.

**Table 1:** Information on education, premorbid IQ and self-rated anxiety/depression at baseline of the patients and controls

	mTBI *n* = 15	OC *n* = 15
Length of education, mean (SD)	12.6 (1.8)	13.3 (1.8)
Estimated premorbid IQ, mean (SD)	99.0 (7.8)	104 (9.8)
HADS-D, median (range)	2 (0–5) (*n* = 14)	1 (0–11) (*n* = 14)
HADS-A median (range)	5.5 (0–10) (*n* = 14)	4 (0–14) (*n* = 14)

Students t-test and Mann–Whitney U-test were used for comparison between the groups. No group differences *where statistically significant*

*OC* Orthopedic controls, *HADS-A* Hospital anxiety and depression scale – anxiety subscale, *HADS-D* Hospital anxiety and depression scale – depression subscale

### Group and time differences in neuropsychological and self-rated variables

As shown in previous articles on this material there were group differences between mTBI patients and OC concerning state fatigue (RPQ-f) and fatigability (DSST-f), at T1 [17]. There was no significant difference in self-rated trait fatigue according to FSS. In the current study we also investigated group differences in total self-rated symptoms (RPQ) and found significant differences between the groups at T1. All differences were in the expected direction at T1, i.e. the mTBI group rated more symptoms and showed more deficits on tests. For the OC group only the item sleep disturbances had a median value that reached 2, indicating current mild problems, all other items had a median of 0. The mTBI group reported mild problems with headaches, noise sensitivity and fatigue, attention problems, forgetfulness and slowed thinking at T1.

At T2 the only statistically significant difference was in total self-rated symptoms. When analyzing the items separately it was noted that headaches and poor memory seemed to be the most persistent remaining problems for the mTBI group, with a median value of 2 (equal to a mild symptom). The OC group had a median of 0 on every item in the RPQ at T2. Both groups improved over time in terms of total post-concussion symptoms and self-rated fatigue.

In order to control for multiple comparisons, the Holm-Bonferroni test was used. The group differences in the overall post-concussion score and objectively measured fatigability remained statistically significant, while the disparities in self-rated fatigue were no longer significant. For more details see Table 2.

**Table 2:** Between group differences concerning post-concussion symptoms, self-rated fatigue and objective fatigability

Measure	mTBI ( T1 *n* = 15, T2 *n* = 13)	OC (T1 *n* = 15, T2 *n* = 13)	U-value	*p*-value (Significance Threshold after correcting for multiple comparisons)	Effect size
**Self-assessment instruments**					
RPQ T1	22 (0–38)	4 (0–15)	40.0	**0.003** (0.008)	0.64
RPQ T2	11 (2–31)	2 (0–18)	27.0	**0.003** (0.01)	0.68
RPQ-F T1	2 (0–4)	1 (0–3)	58.5	0.022 (0.01)	0.50
RPQ-F T2	1 (0–2)	0 (0–2)	52.5	n.s	
FSS T1	4 (2–7)	3 (1–5)	64.5	n.s	
FSS T2	3 (1–6)	3 (1–5)	69.0	n.s	
**Neuropsychological tests**					
DSST-F T1	-1 (-6–4)	0 (-3–6)	58.5	**0.025** (0.025)	0.48
DSST-F T2	3 (-3–9)	0 (-8–7)	69.0	n.s	

Median and range are presented for each variable and group. Results from between group comparison using Mann–Whitney U-test is reported

RPQ is total self-rated symptoms excluding prior symptoms. RPQ-F is self-rated state fatigue excluding prior symptoms. DSST-F is fatigability over time. FSS is self-rated trait fatigue

*OC* Orthopedic Controls, *RPQ* Rivermead Post-Concussion Questionnaire, *RPQ-F* Rivermead Post-Concussion Questionnaire fatigue item, *DSST-F* Fatigability measured with Digit Symbol Substitution Test, *FSS* Fatigue Severity Scale

**Fig. 1:**
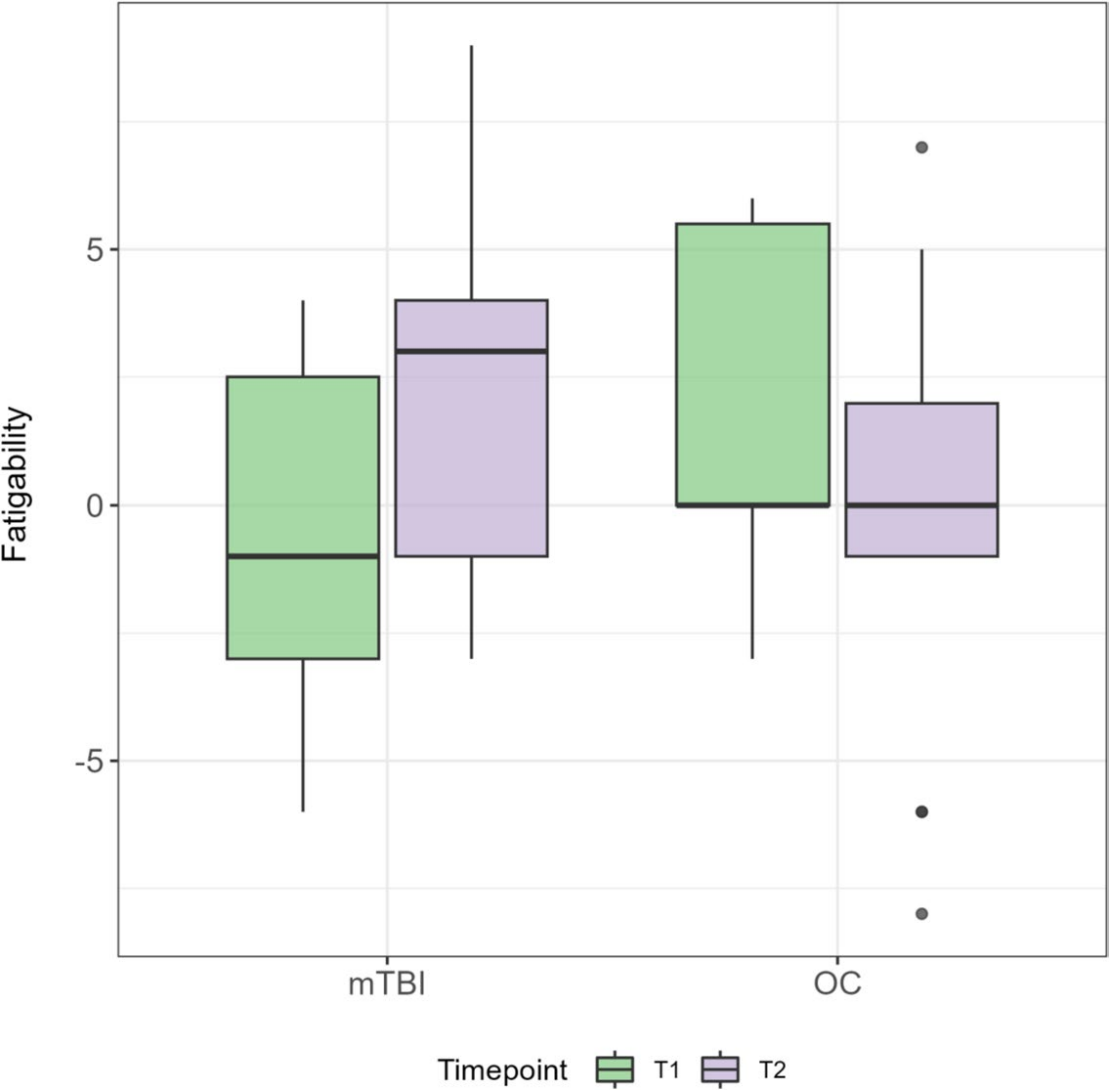
Differences between groups in changes in objectively measured fatigability over time

As shown in Table 3, for the self-rated variables there was a statistically significant improvement over time for all participants (Z_RPQ_ = 97.5, *p*_*RPQ*_ = 0.023; Z_RPQ-F_ = 97.5, *p*_*RPQ-F*_ = 0.030) and no statistically significant difference in change over time between the groups (U_RPQ_ = 65.5, *p* = 0.34, U_RPQ-F_ = 71.5, *p* = _RPQ-F_ = 0.50). Concerning the fatigability variable, no improvement over time could be detected when analyzing all participants taken together (Z = 147.0, *p* = 0.94). However, there was a statistically significant difference in the change over time between the two groups concerning this variable (U = 36.5, *p* = 0.015, effect size = 0.57). As visualized in Fig. 1, the mTBI patients improved over time, while OC group did not change over time concerning objectively measured fatigability.

**Table 3:** Symptom resolution over time concerning post-concussion symptoms, self-rated fatigue and objective fatigability for all participants

Measure (*N* = 26)	T1	T2	Z-value	*p*-value	Effect size
RPQ	11 (0–38)	4 (0–33)	213.0	0.023	0.54
RPQ-F	2 (0–4)	0 (0–2)	97.5	0.030	0.63
DSST-F	0 (-6–6)	1 (-8–9)	147.0	n.s	

Median and range are presented for each variable and time point. Results from between group comparison using Wilcoxon rank sum test is reported

*RPQ* Rivermead Post-Concussion Questionnaire, *RPQ-F* Rivermead Post-Concussion Questionnaire fatigue item, *DSST-F* Fatigability measured with Digit Symbol Substitution Test

### Relationships with cognitive reserve

The two measures of cognitive reserve, years of education and estimated premorbid IQ were highly correlated, Spearman’s rho = 0.673, *p* < 0.001. When analyzing the groups separately the measures were still strongly correlated, although they were slightly lower for the mTBI group compared to the OC group Spearman’s rho_mTBI_ = 0.622, *p*_mTBI_ = 0.013 versus Spearman’s rho_oc_ = 0.700, *p*_oc_ = 0.004.

**Fig. 2:**
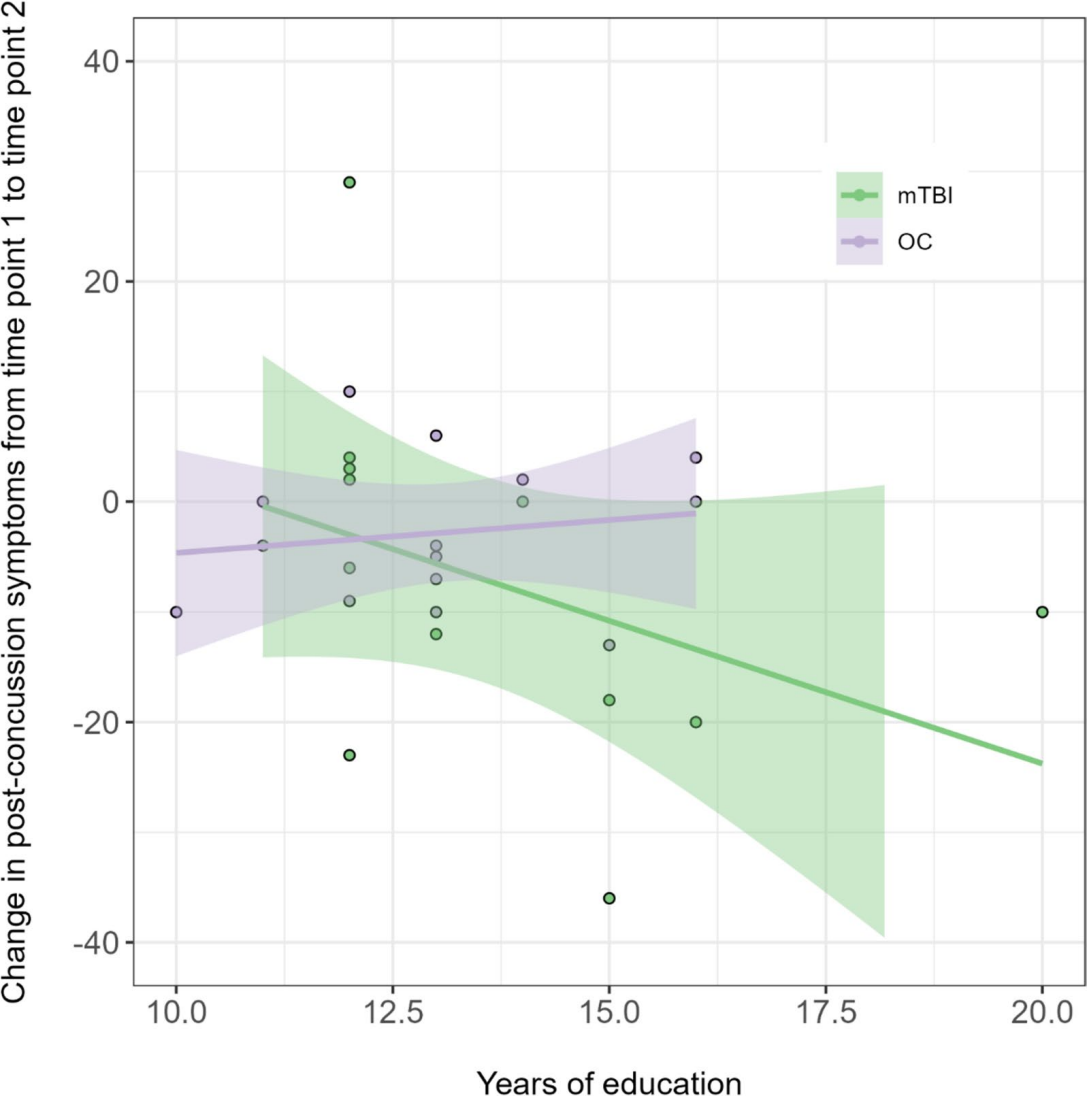
Improvement over time plotted against length of education for both groups

Improvement in RPQ over time (total score) correlated with length of education in the mTBI group, Spearman’s rho = -0.579, *p* = 0.038, but not with estimated premorbid IQ, see Fig. 2. Neither improvement in RPQ-f nor DSST-f correlated with length of education or estimated premorbid IQ for the mTBI group. There were no correlations with improvement over time and length of education/estimated premorbid IQ in the control group on any measurements.

### Resting-state networks

The ICA-analysis using resting state fMRI data sets from both the baseline and the three-month follow-up on patients and controls provided 20 independent components of which 9 were manually classified as artifacts (mainly motion and cardiac/flow artifacts).

### Differences in resting-state networks

**Fig. 3:**
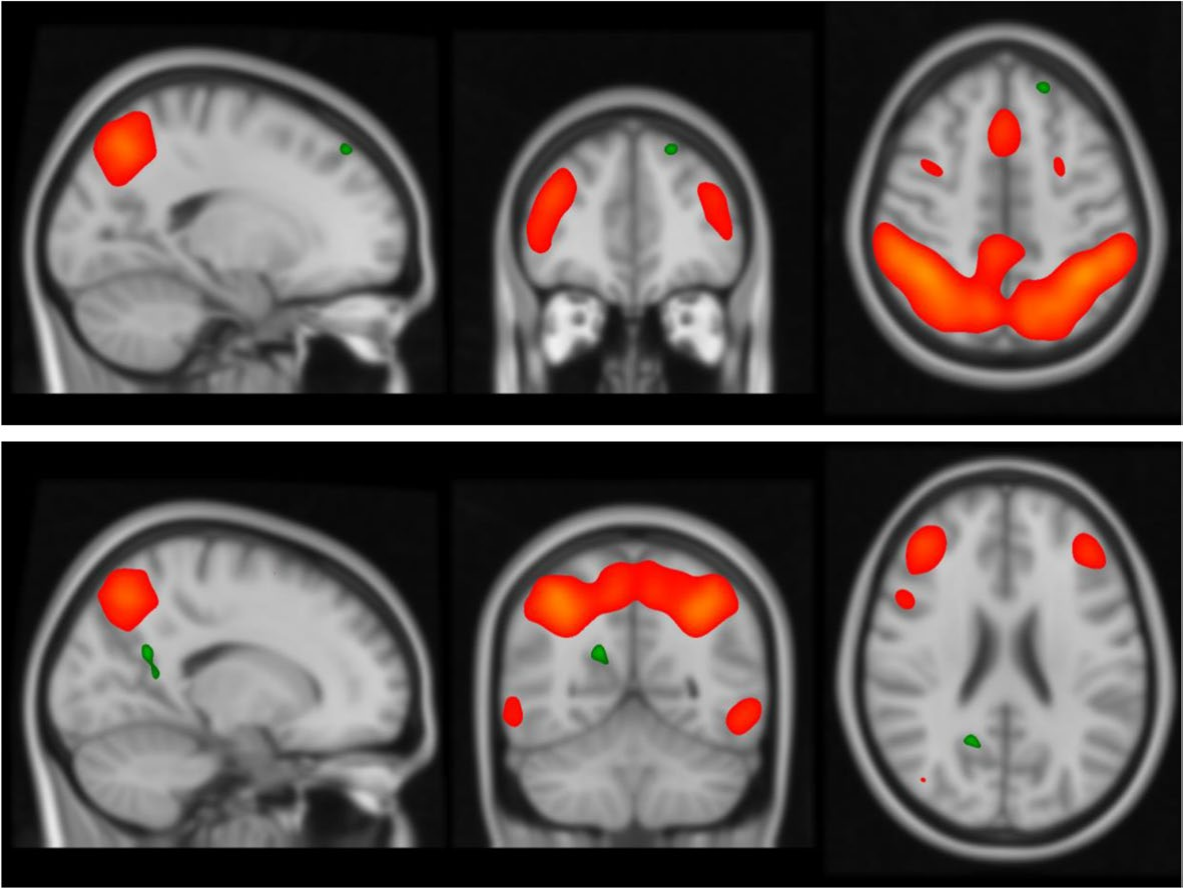
Orange marking reflects the frontoparietal network identified with ICA during rs-fMRI and significant group differences in the left superior gyrus (upper picture) and precuneus (lower picture) are marked green (*p* = 0.05)

Data sets with motion larger than the voxel size (2,4mm) were excluded. In total 3 controls and 4 patients were excluded from the first time point. From the second time point 2 controls and 3 patients were excluded. All the remaining data sets did not show any group difference regarding motion. The group analysis from dual regression revealed significant functional differences in connectivity in a frontoparietal network between the mTBI group and the OC group, see Fig. 3. The mTBI patients showed higher functional connectivity in the left superior frontal gyrus (2 voxels, MNI coordinates 14, -42, 48) while the OC group showed higher functional connectivity in the right precuneus (3 voxels, MNI coordinates -18, 58, 24). The activation differences between groups were only seen when data from both time points were included.

**Fig. 4:**
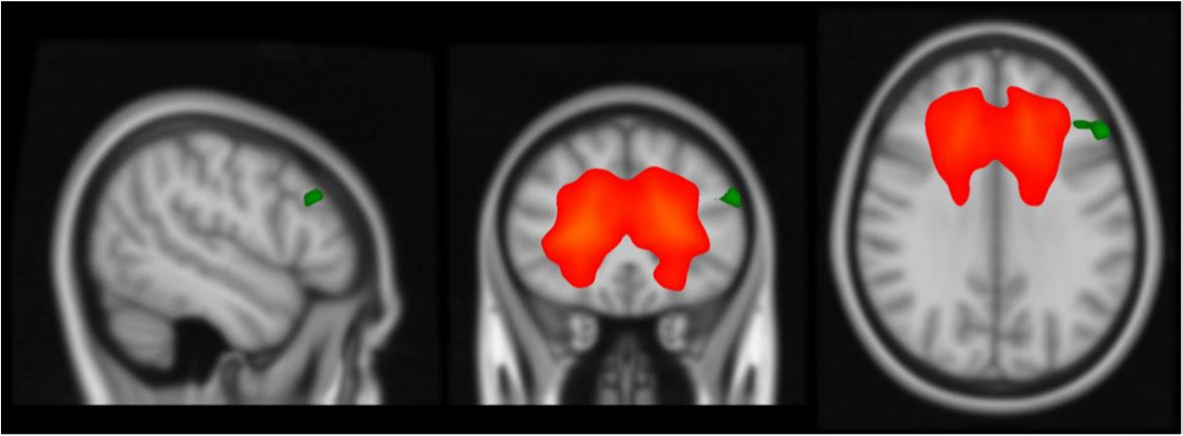
Orange marking reflects the executive control network identified with ICA during rs-fMRI and the significant time differences in the left middle frontal gyrus are marked green (*p* = 0.05)

The time analysis from dual regression showed a difference in IC 1 for the mTBI patients. At T2 the mTBI patients had higher functional connectivity in one cluster in the left middle frontal gyrus (6 voxels, MNI coordinates 42, -30, 32) compared to T1 (see Fig. 4). Dual regression analysis, corrected for multiple comparisons revealed no other significant functional connectivity differences in any of the identified networks over time in either the mTBI group or the OC group separately or combined.

### Relationship between functional connectivity and cognitive reserve/symptoms

No significant correlations were found between measures of cognitive reserve (estimated premorbid IQ and education) and functional connectivity in any of the networks identified at any time points. Likewise no significant correlations were found between functional connectivity and any of the symptom measures.

## Discussion

The aim of the current study was to analyze differences in symptoms and symptom resolution between patients with mTBI and orthopedic controls and the relation to cognitive reserve and brain connectivity. We found that self-rated post-concussion symptoms were still significantly higher in the mTBI group at follow-up, indicating that at least a proportion of the patients with mTBI did not consider themselves fully recovered after three months, as seen in previous studies [35]. Patients with less improvement had lower educational level. In general, there was improvement in the mTBI group concerning slowed thinking, fatigue and noise sensitivity, but mild problems with headaches and poor memory remained.

Group differences in resting-state functional connectivity were found in the frontoparietal network. The mTBI group had increased functional connectivity of the left superior frontal gyrus and decreased functional connectivity of the right precuneus, compared to the control group. The frontoparietal network is believed to be a flexible hub of cognitive control, important for task adaptation and implementation, and often found to be affected in psychopathology and brain injury [36–38]. The superior frontal gyrus is considered to be a part of the dorsolateral prefrontal cortex, which is considered important for higher cognitive functions such as planning, cognitive flexibility, and working memory [39]. Hyperconnectivity after brain injury has been speculated to be a compensatory response of the brain to preserve function despite injury [40]. Hence, an increase in connectivity of this area for patients with mTBI compared to controls could be related to the need to recruit more areas during higher order processing to compensate for injury, as the mTBI group reported more problems concerning attention, memory and slowed thinking. Functional connectivity differences in the left superior frontal gyrus could also be related to more headaches as previous studies have seen a correlation between post-traumatic headache and activity in this area, and this was a persistent problem for the mTBI group [41]. Consistent with this, repetitive transcranial magnetic stimulation to the left dorsolateral prefrontal cortex has been seen to alleviate post-traumatic headache symptoms [42]. The other area where group differences in connectivity were detected, the precuneus, is classified as a rich club node, i.e. one of the most highly integrated network hubs of the brain [5, 43]. Changes in the connectivity of these highly connected areas are commonly observed after brain injury and are thought to reflect the brain’s need to coordinate communication in the brain as it adapts to injuries [43]. A recent article has even been able to link acute changes in connectivity within another rich club node, the thalamus, to persistent post-concussion symptoms, underscoring the significance of examining these alterations in rich club connectivity in order to better comprehend the recovery process after mTBI [44].

Improvement in self-rated post-concussion symptoms correlated with length of education in the mTBI group and plotting the data revealed that the mTBI patients with the lowest level of education rated more symptoms after three months than mTBI patients with higher levels of education. There was no such difference between levels of education and symptom recovery for the OC group. This is in line with previous research indicating that higher cognitive reserve is beneficial for recovery after mTBI, at least concerning cognitive recovery, and that people with lower cognitive reserve are at a greater risk for developing post-concussion syndrome [45–48]. The lack of correlation between education and improvement over time in the control group is most likely related to the fact that this group had relatively few symptoms at baseline and thus no room for improvement over time. Therefore, it is not possible to determine what role educational level plays in symptom reduction for the OC group given the design of the current study.

In the current study there was no significant relationship between a hold test measuring premorbid IQ and recovery according to self-rated post-concussion symptoms. The data, although not significant, pointed in the direction of lower premorbid IQ being related to more post-concussive symptoms. It is possible that the relationship between premorbid IQ and outcome is slightly weaker compared to the relationship between education and outcome in the mTBI group because a Lexical Decision Test might not be an adequate test of premorbid IQ after mTBI. If the patients are suffering from headache, dizziness, etc., they might not be able to put in the effort needed for the test, thereby obtaining lower results. This hypothesis has preliminary support in our data as estimated premorbid IQ and length of education were more strongly correlated in the OC group compared to the mTBI group, although they were still highly correlated in the mTBI group. Another explanation is that educational level might be related to work situation to a greater extent than premorbid IQ. It has previously been reported that manual work is negatively associated with work retention after brain injury [49]. More difficult work retention makes it less likely to be able to adjust and adapt to work demands, thereby potentially negatively influencing symptoms such as fatigue and headache, and people with lower educational attainment might to a greater extent hold a job involving more manual work. There was no correlation between education or premorbid IQ and recovery concerning the fatigue-related variables in the mTBI group or for any of the variables in the control group. Additionally, no significant correlations were found between measures of cognitive reserve and functional connectivity in the brain. However, it is not possible based on this negative finding to rule out any correlation between cognitive reserve and functional connectivity as this study had a relatively small sample, thereby lacking in power to detect such a relationship.

A previous study observing the current patient group approximately 1 week after injury found significant differences between patients with mTBI and minor orthopedic injury regarding objectively measured fatigability and self-rated state fatigue. In the current study we found that these differences did not persist at three months. Nevertheless, concerning objectively measured fatigability we found a significant difference in change over time between the two groups. The mTBI group improved from a level below that of the OC group to a level clearly above that of the controls. For the OC group there was no change over time in this measure. The clear improvement in fatigability for the mTBI group indicates that this measure has the potential to capture cognitive fatigue in the early stages after mTBI, a notoriously difficult task [50]. Furthermore, fatigability is an objective measure of changes in performance, compared to fatigue which relies on measures of self-rated symptoms [51].

Concerning changes in functional connectivity over time the mTBI group showed a difference in functional connectivity related to a network believed to be an executive control network, with more connectivity at follow-up of the left middle frontal gyrus. Increased connectivity over time after injury in resting-state networks has been seen previously and as mentioned above is thought to be related to a compensatory response [40]. Similar to the superior frontal gyrus, the middle frontal gyrus is also a part of the dorsolateral prefrontal cortex [39]. The change in connectivity over time in this area for the mTBI group could therefore possibly reflect changes in compensational connectivity over time corresponding to the reported symptoms diminishing, although not fully recovered. Furthermore, the dorsolateral frontal cortex has been associated with fatigue during task performance [52, 53]. As objectively measured fatigability was the one measure where clear differences in trajectory between groups could be seen, the differences in connectivity over time for the mTBI group could possibly also reflect some of the processes involved in the reduction in fatigability. There were no changes over time in functional connectivity in the control group.

### Strengths and limitations

A strength of the study was that data were collected both at the semi-acute phase and at a more chronic phase of mTBI. Using an orthopedic control group compared to a healthy control group is also an asset since this makes the groups more comparable concerning a recent experience of a mild trauma. Another strength is that self-assessment data, neuropsychological data and imaging data were collected at both time points.

The study has limitations in the form of a small sample size and the fact that a larger proportion of men declined to participate compared to women, which restricts its generalizability. Another limitation is the higher incidence of previous concussions in the mTBI group, suggesting that some of the observed group differences might be associated with a history of prior concussions. Nevertheless, none of the participants with a history of previous concussions reported any lingering symptoms. Furthermore, the time between successive pulse sequences, repetition time, used when collecting the fMRI data was too long. A long repetition time may cause aliasing between resting state networks and cardiac/respiratory signals. However, these artifacts are mainly present in central structures near or within CSF-spaces [54]. Some motion occurred in both the patient group and OC group during the resting state fMRI sequence. Motion correction was performed to minimize the effects of motion and the power spectra along with time curves were controlled for each subject to ensure sure that no motion larger than the acquired voxel size was present. Finally, there is a hardware limitation in using an 8 channel receiver coil causing a limitation in the acquired signal-to-noise ratio. These limitations could affect the sensitivity in detecting functional connectivity differences between the two groups, which are relatively few compared to previous studies [8]. These limitations also prohibited us from properly studying the relationship between not only cognitive reserve, but also symptoms and fatigability, and functional connectivity in the brain in conjunction with recovery after mTBI. Another limitation was the variability in time span, especially at follow-up. However, correlational plots of improvement over time indicated that the variance in follow-up time had a negligible effect on the rate of improvement.

## Conclusions

The current study provides further support for the notion that there is an improvement in post-concussion symptoms over time in a majority of cases after mTBI. However, a small subgroup, possibly related to a lower level of cognitive reserve, seems to have more persistent and even worsening symptoms over time, suggesting that persons with a lower level of education should be more closely monitored after mTBI. The study also revealed functional connectivity differences between patients with mTBI and an orthopedic control group. Larger studies, using more appropriate fMRI methodology, are warranted to confirm the preliminary findings in this study and further explore the relationship between functional connectivity and cognitive reserve as well as symptoms and symptom resolution after mTBI.

## Data Availability

The datasets analyzed during the current study are not publicly available in order to protect the privacy of the participants, in alignment with ethical board requirements. Datasets are available from the corresponding author on reasonable request.

## Abbreviations

BET: Brain Extraction Tool
BOLD: Blood Oxygen Level-Dependent
CT: Computerized Tomography
DMN: Default Mode Network
Dsst: Digit Symbol Substitution Test
Dsst-f: Fatigability measured with Digit Symbol Substitution Test
GCS: Glasgow Coma Scale
FFE: Fast Field Echo
FLAIR: Fluid-attenuated inversion recovery
fMRI: Functional Magnetic Resonance Imaging
FOV: Field of View
FSL: Functional Magnetic Resonance Imaging of the Brain (FMRIB) Software Library
FWHM: Full Width at Half Maximum
HADS: Hospital Anxiety and Depression Scale
ICA: Independent Component Analysis
MNI: Brain template standard developed by the Montreal Neurological Institute
MRI: Magnetic Resonance Imaging
mTBI: Mild Traumatic Brain Injury
RPQ: Rivermead Post-Concussion Symptoms Questionnaire
rs-fMRI: Resting-State Functional Magnetic Resonance Imaging
OC: Orthopedic Controls
TE: Echo Time
TI: Inversion Time
TR: Repetition Time
WAIS: Wechsler Adult Intelligence Scale
